# Remarks on the Diseases Which Prevail in Tennessee during Summer and Winter

**Published:** 1844-02

**Authors:** John W. Richardson

**Affiliations:** Rutherford County, Tenn.


Art. II. *Remarks on the Diseases which prevail in Tennessee during Summer and Winter.* By John W. Richardson, M. D., of Rutherford County, Tenn.*

The diseases of our climate may be divided into two great classes; namely, into Summer and Winter complaints. The chylopoietic viscera, as a general rule, suffer primarily in those of the hot season, as the lungs, the serous and fibrous membranes, are the parts which are most apt to take on disease in the cold season. The diseases of spring are generally a complication of those peculiar to the winter, with some resemblance to those of the warm season; and the diseases of autumn partake much of the character of those of the summer preceding, and, when they continue late, run into the diseases of winter.

Cholera morbus, fluxes of various kinds, gastritis, enteritis, hepatitis, and similar affections, make up the large catalogue of Summer diseases; and they are almost always produced by irregularities, and imprudence in diet or drink; or by exposure to a cool and damp atmosphere, by which the cutaneous secretion is suspended, and the equilibrium of the fluids destroyed. Another great cause of disease is exposure to the sun. The hot season of itself predisposes to the diseases above enumerated, by producing a continued stimulation of the capillary vessels, until they become debilitated by over excitement. The nervous filaments are also debilitated from the same cause—a general *malaise*, with weakness and trembling ensue—a centripetal action is given to all the fluids in a slow and almost imperceptible manner, or else some excitant being thrown into the stomach in the form of indigestible food, acts as an irritant and induces disease rapidly.

---

*Dr. Richardson, as Orator of the Medical Society of Tennessee, read this paper at the meeting of the Society, in May last. In publishing it, we have felt it due to its intelligent author to omit those passages of a popular character which were designed especially for the members of the Society, and for the region in which the address was delivered, but which could not be expected to interest the readers of our Journal.

The most remarkable evidence of disease is the broken balance of the circulation. The equilibrium is destroyed and the burden falls upon the internal organs. Vomiting frequently comes on at this point, and if the malign cause has done no more than simply irritate the stomach, the vomiting ejects the offending substances, an equilibrium is again partially or entirely restored, and, if the patient keep his stomach empty, for a few hours, he gets well. And it is a remarkable fact that those very articles which act as irritants on our stomachs in summer, can be taken with perfect safety in winter. Is it not altogether probable that the process of respiration in cold weather prevents such substances from exerting any deleterious effect upon us? There seems, in fact, to be an absolute necessity for the very articles in winter which we cannot use with impunity in summer. This appears to have some relation to the respiratory function; in other words, to the consumption of oxygen, which is greater in cold than in warm weather. If we may rely upon the facts and reasonings of Professor Liebig, a close connection exists between the quantity of oxygen respired, and the amount of food requisite to maintain the heat and the health of the animal, and a solution is afforded of the well-known fact, that the process of digestion is most vigorous in cold climates and a low atmospherical temperature.

The disease of the greatest moment, and the one which attracts our attention more than any other in summer, is *Fever*. This malady, whose protean forms have excited the curiosity of every man, whose peculiar location has evaded the search of the most diligent, and whose dreadful ravages have baffled the skill of the most learned, still remains a subject of controversy. Fevers exist in every season, and it is very strange to me that fever in the summer and autumn should be called *the* fever, while the same disease in winter should be expressed by some other name; or rather, it is more strange, that fever in the winter should be correctly defined, while fever in the summer is not defined at all. For I affirm, that the article *the*, although used especially to define some particular thing, does not define anything when applied as it commonly is to fever But I may be told we do understand the form of fever when it is defined by the article the. I answer, we do not. Nor can any man prescribe correctly for "the fever." He must first inquire for the symptoms, if he be absent; or, if he be present with the patient, he must examine for himself before he can prescribe correctly.

It is true, the expression, "the fever," usually produces in our minds an association of symptoms which have been generated there by education and observation; but how often is it the case that so much dissimilarity exists, not only in the fevers of different seasons, but also in the fevers of the same season, in different locations, and even in members of the same family, that we cannot prescribe for every case correctly, unless we first see and examine each individual patient. It is very true that many physicians look on every case of fever, arising in the "fever season," as being produced by the same cause, and therefore conclude that the same organs must necessarily be primarily affected, and that a routine practice may be pursued; than which conclusion nothing is more fallacious in theory, or more disastrous in practice.

What gives the peculiarity to fevers? We answer that the season of the year in which the fever arises gives it its greatest peculiarity. Certain seasons, and their vicissitudes predispose certain organs to disease, and it is the disease of those organs which characterises the form of fever. Why, then, are the fevers of the hot season different from those of the cold? We answer, because different organs and tissues predominate in point of disturbance, and give rise to a particular class of symptoms, which in the aggregate are denominated "the fever;" but which require in different cases a modified plan of treatment.

The main question, however, and the one of most difficulty for us to determine is this: *which are the organs most predisposed to disease*, in differsent seasons, *and why are they so predisposed?* As a line of separation can be easily drawn by the close observer between the diseases of the hot and cold sea son, we shall proceed to speak particularly of them; and first, of those peculiar to summer.

As already remarked, the diseases of summer are seated in, and produced primarily by derangement of, some one or more of the chylopoietic viscera, at the head of which stands the stomach. The second in importance is the small portion of the intestinal canal; the third is the liver. And we doubt very much whether there ever was, or ever can be, a summer disease in which one or the other, or all three of these organs are not primarily involved. True, the predisposing cause may operate upon some other part of the system before these viscera are implicated. If, indeed, we except the stomach, which is sometimes promptly disordered by ingesta, the action of the malign cause can never be exerted directly on either of those organs. But that it is in one, or more of them, that the force of the malign cause is spent, and from which all or the most of the morbid effects are radiated, is, we think, entirely demonstrable. And it is also true, that often before we can discover any sign of disease in either of those organs, the whole system has been convulsed by an ague, which is followed by considerable arterial excitement, and all the pains and aches belonging thereto; but in a short time the system relieves itself from this condition, and unless the predisposing cause continue to operate, or the morbid action which was first induced thereby has produced some point of disease, which becomes now of itself a cause, the patient is nearly well. If the predisposing cause be removed, or the patient be removed from its influence, unless some local, organic disease has been induced, the fever will subside of itself.

But in many instances, in summer, we see great derangement of those organs in which the arterial system does not for a time, perhaps not at all, participate, until from considerable ravages every part of the system is affected. And no man pretends to call such cases *fever*. Vomiting, diarrhœa, and cholera morbus are frequent diseases of summer, and how often is it the case that they pass off without inducing a febrile paroxysm? and how often, too, do they usher in the very worst forms of fever? No man can tell whether such a case will terminate in fever. If arterial excitement be superadded to the other symptoms, we call the complaint fever; and here I will remark that the cases which most perplex the physician are those in which a deranged condition of the circulation persists after he has, as he imagines, removed every local cause. But we may be asked, may not this deranged circulation be dependent upon disease of the membrane lining the heart and arteries? We answer yes; and it is upon the correct settlement of this question, in such cases, that will depend the fate of our patient. Memory recalls to my mind some cases of this kind, where the deranged circulation was attributed to an improper source. The *liver* is often charged with it, and a system of purgation is adopted, which, instead of relieving the patient, actually leads to his death. Or this quick, irritable, small pulse, as it is called, may have been produced by improper purging, in the first instance, causing a highly irritated or inflamed condition of the small bowels, in which case the continued use of cathartics will aggravate instead of relieving the symptoms.

The temperature of the summer season is the great predisposing cause of disease. The atmosphere in the hot season becomes warmer and thinner; the whole exterior man is exposed to the increased temperature through the dermoid covering, in which are ramified a large part of the blood vessels belonging to the systemic circulation, and also a proportional part of the nerves. The capillary system of the smaller circulation and its concomitant nerves are also directly exposed to the atmosphere by their ramification through the lungs. Through these two media then, the skin and the lungs, we are directly exposed to the temperature which is peculiar to each season, and also to the vicissitudes of the seasons themselves. It is easy to conceive that some change—some abnormal condition, must be produced in the human system thus exposed.

The transition is often sudden, from heat during the day to a cold damp atmosphere at night. The high temperature causes the capillary vessels to dilate, and also highly excites the nervous filaments; in consequence of which, the blood flows freely to the surface. The low temperature has precisely the contrary effect: the capacity of the capillary vessels is decreased, sometimes very rapidly; centripetal action is given to the fluids, and the internal vessels receive a surplus; plethora of one or more organs is the result, and unless they relieve themselves by increased secretion, as the skin did in the former case, disease is the inevitable consequence. This plethora, or congestion, as it is usually termed, is generally induced in some of the abdominal organs, and the reason why disease is generated in them rather than in the lungs during hot weather is, simply, that the capillary system of the lungs is operated on immediately, and influenced by the same causes and in the same manner that the capillary system of the larger circulation is through the skin; both systems being similarly affected by temperature and its changes. And it is fortunate for us that this is the case, for if the capacity of the pulmonic capillaries could not be increased by temperature, we should be exposed to a class of diseases at a season of the year when, of all others, they would be the most unmanageable, because they would be complicated with chylopoietic derangement. Every practitioner is acquainted with the difficulty of treating thoracic inflammations, especially of the lungs, when they are complicated with irritation or inflammation of the stomach, bowels, or liver. But, fortunately, the most acute, rapid, and dangerous inflammations of the lungs occur at a season of the year when the chylopoietic organs are less liable to take on disease, and we have a single and simple malady to treat.

When the equilibrium is destroyed in the circulation, whether it be produced by exterior causes, or from an excitant within, or by both conjointly, nature generally makes an effort to rid the system of the offending cause by emesis, which she frequently does; and at the same time that the vomiting ejects the irritant from the stomach, it also gives a centrifugal action to the fluids. In this manner the equilibrium is frequently restored, and if the congestion in the internal organs has not already caused local lesion and produced a point of irritation, the patient will in all probability be shortly relieved.

There are other causes eminently productive of disease in the chylopoietic organs, during the hot season: I allude to articles of food and drink. And here I must be permitted to raise my voice against an article esteemed a great luxury in hot weather—I mean ice. Ice never was designed for man in summer as an article of constant use. As a medicine, it is not only a luxury, but a remedial agent of much value. I cannot, however, conceive of a practice better calculated to impair the action of the stomach, than frequent and large draughts of ice-water in hot weather. There is no danger in drinking ice-water in cold weather; no man ever heard of any one's being injured by it. But I honestly believe that much of the disease of our southern cities is produced by this very article, in the hot season. The common water of our springs and wells is sufficiently cold for a healthy man, and even it occasionally produces death very suddenly when taken imprudently. I know that the water in many cities is not only warm, but offensive, and really requires something to make it potable; my advice to the inhabitants of such places would be to use ice water sparingly, if they would pass a healthy summer. Better drink water a little above than below the common temperature of good spring water.

The treatment of summer diseases, and especially of fever, is a subject of great importance. No one has yet given us a correct treatise upon this subject; nor is it probable that we shall ever have one. Not that fever is not successfully treated by many physicians; we know that this is the fact. But, sir, can you sit down and tell of your students how to treat "the fever." If you can, I acknowledge I cannot. Fever is the consequence of a thousand causes, and the great secret in subduing a fever of any form is to ascertain the cause. Remove that and the fever will leave the apartment.

Our remarks upon the treatment of fever must of necessity be very general. The intermitting form of the disease, the simplest of all the complaints of the hot season, is characterized by a chill, head-ache, pains in the back and extremities, frequently vomiting, and then considerable arterial excitement, the continuance of which depends mainly upon the peculiar periodicity of the disease: a copious diaphoresis usually terminates the paroxysm, and the man looks and feels pretty well. The second, third, or fourth day, as the case may be, he has another paroxysm, and at the regular time, a third, and so on. I have seen a man have an ague, followed by high arterial excitement, and profuse perspiration, the paroxysm terminating and again returning in six hours from the attack. This state of things continued for three days, resisting all the ordinary remedial agents, and was finally arrested by giving sixty grains of calomel, and repeating the dose twice in sixteen hours. Each dose procured one evacuation, and only one, from the bowels. The periodicity was completely broken up, and although the agues returned at irregular intervals, they were arrested entirely in a few days by giving sixty grains of calomel every twelve hours. I expect if Dr. Chapman were to hear of this, that he would have me published in every newspaper from the Lakes to the Gulf of Mexico. Well, it made no "holes in his head"—destroyed neither his nose nor his jaws: no, it did not even inflame his gums, nor produce any fetor in his breath.

Do not from this case, however, deem me extravagantly fond of calomel. I am not. I had the mortification, a little more than twelve months since, to see one of my finest children killed by it. She was just past five years of age, and was the subject of the terrible epidemic I described at our last meeting. I gave the medicine myself; three five-grain doses, and one dose of ten grains in the course of forty-eight hours. Her jaws ulcerated—her tongue sloughed, and such a pitiable object I trust never to behold again. So, I present you with two cases—both pictures accurately drawn, and conclude by saying that I never use calomel when other remedies will answer my purpose. When a little will produce the effect, it is wise to use the smallest amount.

The treatment of the intermittent form of fever is usually very simple. An emetic, six hours before the expected paroxysm, and a dose of calomel, rhubarb, and aloes, equal parts, in the form of pills, three hours before the chill, will arrest many cases. If these means fail, sulphate of quinine, taken during the apyrexia, will almost always succeed. The efficacy of quinine in fevers seems to depend not upon any tonic qualities, but upon its febrifuge principle. It prevents the recurrence of a paroxysm by giving a centrifugal action to the fluids, and restoring their equilibrium. It acts principally on the nervous system. Almost every man in the community knows what will cure fever and ague, but the people do not often succeed in it. The reason is this: they neither give the medicine at the proper time, nor in the proper doses. This is the great secret in treating not only intermitting but remitting fevers. Purgatives should always be given in the intermission or remission—about three hours before the expected paroxysm.

The treatment of the Congestive form of fever, as it is commonly called, is more difficult, and requires vigorous and prompt remedies. Many authors who write about this form of fever, I am sure, never saw a case of it; and some even deny the existence of any such disease. It has fallen in my way to see much of it. I treated a great many cases in the fall of 1835, and again in the summer of 1839 and 1840. The paroxysm in bad cases is generally ushered in with great distress in the præcordial region, difficult respiration, attended with frequent and deep sighing, pain in the head and back, vomiting, shrunken features, purgings of various kinds, sometimes like rice water, with green flocculi floating in it—these flocculi are nothing but mucus when you wash off the green tinge. Again, I have seen as much as half a gallon of reddish serum thrown off at once; great thrist attends with restlessness, and a constant desire for fresh air. Reaction comes on after a while, slowly, and is generally very partial. In the course of twelve hours the patient becomes tolerably comfortable, and if you did not know what had been his condition, you would say there was not much the matter with him.

But the next day would disclose to you the alarming state of things. You may succeed in getting him through the second paroxysm, but if you have not afforded him relief before the third supervenes, you have hardly anything to hope.

I should be glad to give you the treatment of such a case in a stout healthy man, and also in a pregnant female, but it would be extending this paper too far. I may do so on a future occasion.

The next, and most important, inquiry is, how are those attacks of fever to be prevented? If miasmata be admitted the exclusive cause of fever, I have nothing new or interesting to communicate. But I am certain that these agents are charged with a great deal too much. I have practised medicine for eleven years, during which time my practice has been extended over every peculiarity of location to be found in Rutherford county: on creeks, on the river, on frog-ponds, and mill-ponds—over the rich alluvial soil common to our water-courses, and on the dry, gravelly oak-ridges removed from the river, and among the rocks and cedars where a domestic hen could scarcely find leaves enough to make her nest; and I declare that I have seen as bad forms of fever in one district as in the other. I have seen the worst forms of fever pervading the high lands during the hot season, when the people on the river and creeks were comparatively healthy. I am not prepared to deny the malarious origin of our summer and autumnal diseases. I know those diseases are very common in districts known as malarious; but may they not be produced by other causes, independent of miasmata? The atmosphere undergoes great changes, sometimes very rapid changes, in such districts in the course of twenty-four hours, as respects density and temperature. Considerable impression must be made on the extremities of the sanguiferous and nervous systems, and here the first impression is usually observed from the action of external malign causes. But I forbear going into a discussion of this subject.—The preventives consist in as little exposure to the sun as possible, and when exposed, moderate labor is better than idleness; the night air should be avoided; and if you are compelled to ride, put on some additional clothing, and take a little light nourishment. The diet should be vegetables, chiefly—stomach never to be loaded with anything—after labor, or exercise, care to be used not to cool off too rapidly—fires to be kindled in our houses, every morning and evening, and ice water to be avoided. These precautions being observed, my experience and observation authorize me to say, that we can live in "the malarious district" and escape "the fever."

Another disease peculiar to the hot season, is Cholera Infantum. This is indeed a tangible something—a disease, about which we can understand each other. Cholera infantum or "the summer complaint," is a disease peculiar to children in the hot season, provided they are of, or near a certain age. When I say that it is peculiar to children of a certain age, and during the hot season, I do not mean to be understood as conveying the impression that children will have cholera infantum, in the summer, in any event. I mean simply, that the offending causes from without and within the system, are peculiarly apt to induce cholera infantum in children of a certain age, during the warm season. Children who are born in the winter or spring, generally pass through the first summer without any inconvenience from this disease. Two causes are mainly instrumental in producing it. The increased temperature is the great *predisposing* cause, but improper clothing and feeding are the two fruitful agents in bringing on the attack. I am aware that *dentition* is usually assigned as the cause, but this idea always seemed irrational to me, and to be founded in fact upon erroneous physiology. It is true, that cholera infantum is most common with children during the period of dentition, but then I cannot adopt a philosophy which charges a naturally healthy process with the production which those distressing and dangerous diseases which befall children at that period. The true reason why this disease generally appears in children of a certain age and during the hot season, has always seemed to me to be this:—Children are usually weaned between the ninth and the eighteenth month; dentition usually commences before this period and continues beyond it. The change in the diet of children is a fruitful exciting cause. They are seldom fed on such articles as they ought to have, and their food is usually badly prepared, and the feeding succeeds the weaning too suddenly. Solid articles of food, and often of an indigestible character, are given to them before they are able to masticate—they swallow them in the solid form—an irritant is thrown directly into the stomach, to run the whole course of the digestive tube, and finally to be thrown off in the same state in which it was received. Children, while being weaned, are treated after this manner four or five times a day, and then often put to bed at night with a piece of cake in their hands.

Improper clothing is another cause of cholera infantum. Children are easily impressed by atmospheric vicissitudes, if they are not attended to, and their clothing such as will keep up an harmonious action between their systems and the change without. A good maxim, then, for old persons, as well as for children, is to change their clothes with the changes of the weather, and always to err, if either way, on the side of too much, rather than too little clothing. Children during this period are generally too thinly clad, and their clothing is scarcely ever adapted to the vicissitudes of our climate.

The disease, at the onset, is easily managed. An emetic to empty the stomach, if it has not already been emptied sufficiently, and a brisk calomel purgative, if the bowels have not been actively opened, will relieve the majority of cases. But if the complaint is of several days' standing, it frequently becomes serious, and quite intractable, unless treated with judgment as well by the nurse as the physician. Alterative portions of calomel, combined with prepared chalk and ginger, I have found an excellent remedy, given in small doses, and at short intervals; allowing the child but little to eat or to drink. The warm bath to invite the fluids to the surface of the body, and, if there is much inaction in the capillary vessels, a flannel shirt are to be adopted. I know this is contrary to the views of many physicians, but I am able to sustain it against any attack, let it come from what quarter it may. A flannel shirt, in the month of August, sounds like an absurd remedy to many ears, but experience confirms its remedial virtues in chronic cases of cholera infantum. My advice to mothers would be either to wean their children before the warm season commences, or else, if practicable, to postpone it till cold weather.

Here my remarks upon the diseases of the hot season must close. Without exhausting the subject, I have said enough to afford an outline of the causes which produce them, and of the treatment which I have found effectual in relieving them. The same general remarks will apply to all our summer diseases.

The complaints of winter are the next in order, having already premised that those of autumn and spring are of a mixed form, partaking of the character of those of the summer and winter preceding, and inclining also to those which are shortly to follow.

The diseases of winter are principally confined to the lungs and their appendages. I have already alluded to the distribution of the capillary vessels through the lungs, and endeavored, briefly, to show the reason why the lungs are not so much disposed to take on disease during the hot season, as the abdominal viscera. This I attempted to account for by the fact, that the capillaries of the lungs are immediately and directly influenced by the increase of temperature equally with the capillaries of the skin. The process of respiration carries to them every vicissitude of temperature which the atmosphere experiences. Another reason why they do not suffer equally with the abdominal viscera, is to be found in the comparative shortness of their veins, and the activity of their circulation. Still the lungs are liable to disease; but I would remark that no viscus in the great cavities is more easily relieved of inflammation than the lung, provided there is no predisposition in it to tubercle. The treatment is simple, and must be prompt, decisive, and energetic.

I would revert to the subject of fevers for one moment, for the purpose of remarking, that during the stage of congestion the lungs are seriously implicated, as is evidenced by the tightness across the chest, the difficulty of breathing, and other symptoms. This state of things, however, ordinarily passes away with the particular stage of the fever, and the lungs sustain no lasting injury. But this is not always the case. If tubercles exist in the lungs they are rapidly developed by the febrile commotion, and the agues are followed by phthisis pulmonalis. Thus, it has been the remark of all physicians in this region of the State, that the cases of consumption were greatly multiplied after the sickly summers of 1839 and 1840.

The only other point belonging to the subject which I shall stop to discuss is, why are the lungs and their appendages, and the serous, fibrous, and fibro-cartilaginous membranes generally, more predisposed to disease in winter than they are in summer? That pleurisies, pneumonias, catarrhs, rheumatism, &c., are peculiarly diseases of winter, is a fact which no one will question. But why is it, that while exposure, in summer, to the night air, induces affections of the abdominal viscera, exposure to the same cause in winter, will induce disease of the thoracic organs, or an attack of inflammatory rheumatism? These diseases are regarded as essentially inflammatory; no controversy can exist as to their pathology. Why they are the products of cold weather, especially, is a question which it may be hazardous to attempt to answer; but if I were to venture an opinion concerning the cause, I should say that it was connected with the large amount of oxygen inspired, and of carbonic acid exhaled in a cold atmosphere. But, whatever may be the latent source of these maladies, the concurrence of the profession relative to their treatment is very decided, and leaves but little to be said on this point. Every practitioner understands that the measures demanded are the antiphlogistic—the lancet, tartar emetic, calomel and opium.

May 1, 1843.

---

[At the conclusion of Dr. Richardson's Address, a member of the Society, in reply to the allusion to Professor Chapman, remarked that the friends of that distinguished teacher had disavowed for him the authorship of the ridiculous paragraph, which went the rounds of the newspapers, about a year since, describing, in exaggerated terms, the injuries resulting from the employment of calomel. As the allusion is retained in the paper now published, this statement seems to be due to all the parties concerned. Y.]

